# Exoskeleton gait training with spinal cord neuromodulation

**DOI:** 10.3389/fnhum.2023.1194702

**Published:** 2023-05-11

**Authors:** Yury Ivanenko, Elena Y. Shapkova, Daria A. Petrova, Daria F. Kleeva, Mikhail A. Lebedev

**Affiliations:** ^1^IRCCS Santa Lucia Foundation, Rome, Italy; ^2^Saint-Petersburg State Research Institute of Phthisiopulmonology, Saint Petersburg, Russia; ^3^Institute of Translational Biomedicine, St. Petersburg State University, Saint Petersburg, Russia; ^4^Vladimir Zelman Center for Neurobiology and Brain Rehabilitation, Skolkovo Institute of Science and Technology, Moscow, Russia; ^5^Faculty of Mechanics and Mathematics, Lomonosov Moscow State University, Moscow, Russia; ^6^Sechenov Institute of Evolutionary Physiology and Biochemistry of the Russian Academy of Sciences, Saint Petersburg, Russia

**Keywords:** exoskeleton, gait, spinal cord injury, neuromodulation of spinal networks, neurorehabilitation

## Abstract

Neuromodulating the locomotor network through spinal cord electrical stimulation (SCES) is effective for restoring function in individuals with gait deficits. However, SCES alone has limited effectiveness without concurrent locomotor function training that enhances activity-dependent plasticity of spinal neuronal networks by sensory feedback. This mini review discusses recent developments in using combined interventions, such as SCES added to exoskeleton gait training (EGT). To develop personalized therapies, it is crucial to assess the state of spinal circuitry through a physiologically relevant approach that identifies individual characteristics of spinal cord function to develop person-specific SCES and EGT. The existing literature suggests that combining SCES and EGT to activate the locomotor network can have a synergistic rehabilitative effect on restoring walking abilities, somatic sensation, and cardiovascular and bladder function in paralyzed individuals.

## Introduction

By activating the locomotor network, spinal cord electrical stimulation (SCES) can be utilized to restore gait in patients who have suffered from the effects of spinal cord injuries (SCI) (Megía García et al., 2020). This method is of particular interest for modulating neuronal circuits enacting locomotion because the executive component of the locomotor pattern generation circuitry and the “common final pathway,” that is motoneurons, are located in the spinal cord (Grillner and El Manira, 2015). By targeting this circuitry, SCES has the potential to promote activity-dependent plasticity, which can improve the effectiveness of rehabilitation. However, SCES may not be as effective when used alone as when it is combined with gait training, such as exoskeleton gait training (EGT).

Powered exoskeletons have been extensively developed to offer new opportunities for people with significant gait impairments (Swinnen et al., 2010; Sale et al., 2012; Onose et al., 2016; Sanchez-Villamañan et al., 2019; Stampacchia et al., 2022). New control strategies such as brain-machine interfaces and spinal cord neuromodulation can improve the development and use of exoskeletons (Lebedev and Nicolelis, 2011; La Scaleia et al., 2014; Vouga et al., 2017; Gill et al., 2018; Benabid et al., 2019; Rowald et al., 2022).

In this review, we focus on the treatment approach that combines EGT with neuromodulation of the spinal pattern generation networks using SCES. We evaluate the available evidence for the benefits of combined interventions. Given that SCI is a heterogeneous condition and that the spinal cord is a neural structure capable of plastic changes, a special emphasis is placed on the role of functional assessment of the spinal circuitry for developing personalized EGT + SCES therapies. We consider recent research that utilizes this approach to investigate the foundations of such interventions, the suitability of combining EGT and SCES treatments for individuals with SCI, and the potential for therapeutic benefits.

## Exoskeleton gait training

In recent years, significant advancements have been made in the development of wearable powered exoskeletons designed to aid in walking and promote motor recovery. This has led to the creation of several solutions for gait assistance and rehabilitation (Onose et al., 2016; Sanchez-Villamañan et al., 2019). Exoskeletons have been shown to enable overground weighted walking and gait training in humans with paralysis (del-Ama et al., 2012; Sylos-Labini et al., 2014b; Shapkova et al., 2020). In the last decade, a variety (>50) of exoskeletons have been designed with various practical requirements to ensure safety, postural stability, appropriate output torques and long time performance, compliant actuation and mechanisms, cost-effectiveness, size adjustability, effectively wearable and psychologically acceptable (Sanchez-Villamañan et al., 2019). Specific requirements for a complete paraplegia and for effective interaction with the user have also been developed based on a series of exoskeletons clinically tested in small groups of subjects (Onose et al., 2016; Scivoletto et al., 2019). However, there is still an ongoing debate regarding the effectiveness of EGT in individuals with SCI who are severely paralyzed (Contreras-Vidal et al., 2016; Fisahn et al., 2016; Scivoletto et al., 2019).

Despite significant progress in exoskeleton technology in recent years, fully functional and optimal assistive device still have to emerge from this research. Moreover, there are still challenges that need to be addressed before lower limb exoskeletons can be utilized in practical settings (Onose et al., 2016; Sanchez-Villamañan et al., 2019). In addition to the technological complexity and locomotor capabilities of exoskeletons, it is essential to understand the interactions between humans and exoskeletons and the long-term sensorimotor adaptations that occur while walking in the exoskeleton. Although understanding these interactions is crucial for making EGT effective, physical interactions between humans and exoskeletons have not been thoroughly studied in the available literature (Massardi et al., 2022). The existing literature mostly addresses generic kinematic and kinetic indicators of exoskeleton-assisted gait (Pinto-Fernandez et al., 2020), which allows making only limited conclusions about the neural control strategies and adaptations. For instance, substantial changes in the muscle coordination patterns could occur during exoskeleton walking in both patients and neurologically intact individuals (Moreno et al., 2013; Sylos-Labini et al., 2014b). An abnormal spatiotemporal integration of activity in specific spinal segments could also occur and result in a risk for failure or abnormalities in gait recovery (Ivanenko et al., 2009), especially when using exoskeletons for gait training in young children during their critical developmental periods of spinal locomotor network maturation (Cappellini et al., 2020). Therefore, establishing benchmarking performance indicators that evaluate the effect of walking exercises using exoskeletons on spinal plasticity is crucial for the successful integration of exoskeletons into the gait rehabilitation process (Zhvansky et al., 2022). The development and usage of exoskeletons can benefit from appropriate procedures and metrics for their evaluation (Pinto-Fernandez et al., 2020; Zhvansky et al., 2022) as well as from implementation of complementary treatments that modulate the locomotor circuits, such as SCES.

## Exoskeleton gate training combined with spinal cord electrical stimulation

The growing interest to restoring and enhancing gait control in individuals with neurological disabilities has led many researchers to pursue innovative approaches that aid in activating the spinal circuits for locomotion. A significant method for facilitating locomotion involves neuromodulation of the central pattern generation (CPG) circuitry with the stimulation of the spinal cord. It has been shown that stepping-like movements in humans can be elicited when spinal locomotor circuits are activated by relatively non-specific stimuli such as vibratory stimulation of muscle receptors (Gurfinkel et al., 1998), electrical stimulation of the peripheral nerves (Selionov et al., 2009), electromagnetic stimulation of the spinal cord (Gerasimenko et al., 2010), or transcutaneous or epidural SCES (Shapkova, 2004; Gerasimenko et al., 2015; Hofstoetter et al., 2018). All these techniques mostly act on the large-diameter afferent fibers entering the spinal cord through the dorsal roots, which eventually project to several segments of the spinal cord. The latter two methods (and especially epidural SCES) are particularly powerful due to the proximity of the electrodes to the dorsal roots and therefore due to a larger number of the total afferent fibers being activated. Historically, stimulation of the afferents was one of the first methods for inducing cyclic limb movements in animals (Roaf and Sherrington, 1910), and similar approaches for activating spinal CPG circuitry are currently being employed using animal models (Lavrov et al., 2015; Wagner et al., 2018). Epidural spinal cord stimulation is an invasive method that requires medical justification and a neural surgery, which limits its applicability for gait restoration in SCI individuals. Transcutaneous SCES is less selective, but it activates similar neural structures (Hofstoetter et al., 2018). This method being non-invasive technique brings additional benefits in some cases (Gerasimenko et al., 2015). We will not provide a comprehensive overview of the history of spinal cord neuromodulation in this review and instead refer readers to other sources. Nevertheless, it is important to emphasize that SCES is a critical technique for altering the functional state of the spinal cord and promoting improvements in function and health for individuals who are severely paralyzed.

What are the benefits of combining SCES with EGT? While EGT provides a unique opportunity for SCI patients to experience overground weight bearing stepping and to be used in real walking scenarios, its effectiveness in severely paralyzed individuals is very limited (Contreras-Vidal et al., 2016; Fisahn et al., 2016; Scivoletto et al., 2019). On the other hand, the effectiveness of SCES alone (e.g., in the supine or sitting position) is also limited and needs to be complemented by real locomotor training. It is becoming increasingly clear that the spinal cord is not a rigid neural network that mostly relays information between the spinal and supraspinal networks. Instead, it is capable of undergoing plastic changes, some of which could be long lasting or even irreversible. The spinal neurons also multiplex information as the same motoneuron or interneuron could participate in a vast repertoire of possible movements. Inhibitory synapses prevail in the spinal cord (Levine et al., 2014) and serve to maintain the network’s stability and enable an appropriate involvement of spinal reflexes in various motor tasks. Risks increase for abnormalities in the spinal CPG circuitry and consequently failure of gait recovery (Ivanenko et al., 2009) because of the abnormalities in spatiotemporal integration of activity in specific spinal segments and turnover or formation of new synapses in the spinal interneurons, where the half-life of synaptic proteins can be on the order of hours to weeks (Marder and Goaillard, 2006). Therefore, spinal cord recovery could improve if appropriate task-dependent activation is achieved of sensorimotor circuits using a combination of EGT and SCES.

Walking in an exoskeleton while SCES is applied allows coupling of continuous sensory feedback with activity-dependent plasticity of spinal neuronal networks, which represents a great therapeutic potential. The exoskeletons are designed to bear the full body weight and provide full loading of the body weight on the feet during the stance phase of assisted walking. It is also worth noting that overground EGT typically involves walking with crutches (since the exoskeleton cannot provide full balance) and allows to use the upper body and arm muscles to assist leg movements and balance control by coordinating arm, trunk and lower limb motion and thus promoting connections between lumbosacral and cervical enlargements (Sylos-Labini et al., 2014a; Gad et al., 2017; Shimizu et al., 2017). To engage further interlimb networks during EGT, interlimb modulation of spinal motor excitability can probably be evoked by bidirectional transcutaneous SCES over the cervical and lumbosacral enlargements (Atkinson et al., 2022), although the favorable results of such medical treatment still need to be investigated. Finally, both EGT and SCES could improve autonomic function in SCI patients (Aslan et al., 2018; Legg Ditterline et al., 2021; Squair et al., 2021; Stampacchia et al., 2022), so that their synergistic therapeutic effect could yield benefits for multiple functions, in addition to the locomotor function.

Neuromodulation of the spinal cord appears to be beneficial for promoting the locomotor function and could result in some immediate effects, within a single day, on motor activities after motor complete paraplegia (see, for instance, Gill et al., 2018; Rowald et al., 2022). The rationale behind a combined EGT and SCES treatment corroborates a general notion regarding the facilitatory outcome of SCES on the functioning of the locomotor pattern generation circuitry when applied in the rest position or using manual gait assistance or robotic devices for walking on a treadmill (e.g., Shapkova, 2004; Gill et al., 2018; Hofstoetter et al., 2018; Wagner et al., 2018; Siu et al., 2022). EGT enables more autonomous gait performance, postural control, full limb loading and whole-body coordination, and may facilitate a long-term training of adaptive gait to support everyday mobility in people with SCI.

Only a limited number of recent studies have investigated the potential benefits of combining EGT and SCES treatments in individuals with SCI, and these studies have shown promising results. Individuals with SCI who are new to using a powered exoskeleton need to undergo training consisting of several sessions in which they learn to walk with crutches while wearing the exoskeleton (including standing up and sitting down, initiating walking, adapting to and controlling walking speed, turning, etc.) before they can receive the combined EGT and SCES treatment (Shapkova et al., 2020). This treatment approach involves activating the central pattern generation (CPG) circuits, which heavily rely on a consistent excitatory drive (which can be achieved through SCES with or without specific pharmacological neuromodulation) combined with step training using an over-ground exoskeleton. In particular, Gad et al. (2017) in a case study reported that transcutaneous SCES combined with pharmacological treatment enhanced the level of effort and improved the coordination patterns of the lower limb muscles, resulting in a continuous stepping motion in the exoskeleton along with the improvements in autonomic functions including cardiovascular and thermoregulation. In a large cohort of SCI patients, Shapkova et al. (2020) showed that percutaneous SCES of the lumbar enlargement and exoskeleton-induced gait worked together well to assist walking in SCI patients. Additionally, anti-spastic stimulation of the spinal cord at high frequency enabled individuals with severe spasticity to be able to use the exoskeleton for walking. While most individuals with SCI did not completely abandon their wheelchair use after the training, this study demonstrated that a 2-week intensive program of EGT while simultaneously receiving SCES significantly enhanced locomotor ability, improved compensatory sensitivity (including non-differential sensations of passive leg joint motion and a feeling of support), and improved neurological signs in individuals with chronic SCI, even those with complete paralysis (Shapkova et al., 2020). In addition, two participants who were previously unable to walk started to walk using walkers and ceased using their wheelchairs indoors, which was a significant change in their daily routine. The use of EGT in individuals with severe paralysis may not only be beneficial for assisting with walking but also for rehabilitation when combined with SCES. All participants observed a facilitation of stepping with SCES, so the results of these studies are encouraging. However, more research is need as the effects were limited, should be validated in a larger population of patients with SCI, and could vary due to various clinical and methodological factors.

## Individual differences and responsiveness of pattern generation circuitry to SCES

The other important aspect for developing locomotor and CPG-modulating therapies is physiologically relevant assessment of the state of the spinal circuitry when using person-specific SCES. The rationale is the following. The spinal cord normally possesses a notable degree of plasticity (Ivanenko et al., 2017), redundancy among the locomotor network (Pham et al., 2020) and even atrophy in the spinal circuitries distal to the lesion (Dietz and Müller, 2004). If the state of the spinal circuitry is changed or impaired, it is controlled in a specific way by the descending motor pathways, which in turn results in a specific involvement of the supraspinal structures controlling the spinal neurons. Such reciprocal spinal–supraspinal compensatory mechanisms could create a risk of irreversible changes in the state of the locomotor circuitry in some pathologies (Friel et al., 2014) and could produce some difficulties for the developments of the therapeutic procedures restoring locomotor function.

To effectively use CPG-modulating therapies, it is crucial to understand how to evaluate the functional state of the spinal network and its response to modulatory inputs. Since SCI affects people differently, it is important to examine and study the distinct variations in the spinal cord condition. Some research has been done to analyze the individual physiological state of spinal multisegmental reflex pathways in relation to CPG-modulating therapies (Dy et al., 2010; Thompson and Wolpaw, 2014). Even in neurologically intact individuals, the effects of sensorimotor neuromodulation manifest themselves only in portion of subjects (Selionov et al., 2009; Gerasimenko et al., 2010), and the high individual responsiveness of pattern generation circuitry to tonic sensory input in both the upper and lower limbs appears to be related to enhanced stretch reflexes (Solopova et al., 2022). In SCI patients, there is a relationship between the facilitation of segmental reflexes and the ability to recover gait (Dietz et al., 2009). Additionally, gait training has been shown to bring the stretch reflex excitability to normal (Shapkova, 2004; Thompson et al., 2013) and the active engagement of reflex operant conditioning has been used to rehabilitate locomotor function (Thompson and Wolpaw, 2014). Also, the extent has not previously been exploited to which the modular organization of locomotor networks and its impairment in SCI (Ivanenko et al., 2013; Sun et al., 2022) are related to the rhythmogenesis of the spinal cord. Therefore, individual differences in the state of the spinal locomotor circuitry and further methodological developments of its assessment are essential as markers of the individual response to different stimuli or treatments.

The specific procedures used to stimulate the spinal cord and to train SCI patients are instrumental for activation of the spinal CPG network and its restoration. In several studies, neuromodulation tools have been developed and tested in both neurologically intact individuals and patients. For instance, non-invasive SCES has become widespread for both basic and clinical research (Gerasimenko et al., 2015; Mayr et al., 2016; Solopova et al., 2017). The effectiveness of different stimulation parameters has been explored (Iwahara et al., 1992; Dimitrijevic et al., 1998; Shapkova et al., 2020). While SCES represents an effective method for activating the spinal motor pools, it is important to note that SCES is rather non-specific and activates a variety of inputs. These inputs aim at reinforcing synaptic connections of the spinal pattern generation circuitry below the lesion and transforming it to a physiologically active state. Yet, the spinal network are multifunctional and that the same interneurons and motoneurons participate in a different motor actions. Additionally, spinal segments are differently involved in specific locomotor tasks where the activity of motor pools is typically patterned into bursts with phase specificity (Ivanenko et al., 2008). We have previously shown that these temporal patterns correlate with global kinematic goals of locomotion, and that in the future they could be useful to drive neuroprostheses for SCI patients that utilize spatially distributed stimulators (Ivanenko et al., 2003). This approach has been successfully implemented in recent studies using both epidural (Rowald et al., 2022) and transcutaneous (Siu et al., 2022) multi-segmental SCES to restore leg motor functions after complete paralysis. They aimed at arranging the optimal position of electrodes and configuration of activity-specific stimulation programs that reproduced the natural activation of motor pools underlying each activity. Finally, reflex-related activity of lower limb muscles innervated from the spinal segments below the lesion could also be beneficial for gait rehabilitation. Assisted walking in the exoskeleton, when it is combined with SCES, provides the engagement of natural sensory stimulation that facilitates the expression of locomotion, permits its adaptation to the environment, and helps to induce use-dependent plasticity, which is a hallmark of physical therapy treatment (Brumley et al., 2017).

## Concluding remarks

Combining SCES with EGT is an efficient approach to rehabilitation of patients paralyzed after SCI. This method enacts sensorimotor functions while increasing the activity in spinal networks. Although EGT and SCES techniques have advanced significantly in recent years, further improvements are still needed, particularly in understanding how SCES modulates the excitability of spinal circuitry. Although promising immediate effects of SCES in enabling voluntary control of leg movements, more research is needed to determine its effects when combined with EGT, as well as long-term effects of this treatment. To develop clinical treatments, a range of factors should be considered, including individual differences in spinal cord physiology, personalized CPG-modulating therapies during EGT, and accumulating evidence of their therapeutic potential.

## Author contributions

All authors listed have made a substantial, direct, and intellectual contribution to the work, and approved it for publication.
